# COVID-19 Vaccination in Cancer Patients: A Review Article

**DOI:** 10.1177/10732748221106266

**Published:** 2022-09-06

**Authors:** Rana Mekkawi, Bassant A. Elkattan, Alaaeldin Shablak, Mohammad Bakr, Mohamed A. Yassin, Nabil E. Omar

**Affiliations:** 1 Independent researcher; 2 Department of Medical Oncology, National Center for Cancer Care and Research, 36977 Hamad Medical Corporation, Doha, Qatar; 3 Department of Clinical Hematology, National Center for Cancer Care and Research, 36977 Hamad Medical Corporation, Doha, Qatar; 4 Department of Pharmacy, National Center for Cancer Care and Research, 36977 Hamad Medical Corporation, Doha, Qatar

**Keywords:** antiCD20 therapy, cancer immunotherapy, cellular therapy, coronavirus disease 2019, hematologic malignancies, hematopoietic stem cell transplantion, radiotherapy, solid tumors, targeted therapy, vaccines

## Abstract

Coronavirus disease 2019 (COVID-19) infection is caused by severe acute respiratory syndrome coronavirus 2. Adults with cancer are immunocompromised due to several causes including cancer itself and immunosuppressive therapy. Thus, cancer patients are more susceptible to develop COVID-19 infection. As COVID-19 vaccines became available, patients with cancer would benefit from receiving the vaccine. This article aims to review the recent evidences and recommendations about COVID-19 vaccination in cancer patients.

Current guidelines recommend that patients with cancer should have the priority to receive the vaccine given their immunocompromised state. The timing of administration varies depending on cancer type and treatment. Generally, the vaccine should be given before starting the chemotherapy if possible or in between chemotherapy cycles and away from nadir phase. For other cancer treatments, it is recommended to give the vaccine when there is evidence of blood count recovery. In general, induction therapy and treatment for newly diagnosed patients should not be delayed for the vaccination purpose. It is noteworthy to mention that cancer patients especially those with hematologic malignancies might have absented or attenuated response to the vaccine due to their pathophysiological status.

On the other hand, the current vaccine guidelines have been criticized for lacking evidence on some important topics that need to be addressed. Firstly, some vaccines have been granted an emergency use authorization, prior to the usual comprehensive safety and efficacy evaluation process. Secondly, specific populations including cancer patients were excluded from the approval trials for safety reasons. Finally, some recommendations regarding the COVID-19 vaccines are extrapolated from other vaccines studies. Further studies are required to fill these gaps and observational studies that include cancer patients are warranted to have a better understanding of the safety and efficacy of the vaccines in cancer patients.

## Introduction

Severe acute respiratory syndrome coronavirus 2 (SARS-CoV-2) is the strain of coronavirus that causes coronavirus disease 2019 (COVID-19) infection, the respiratory illness responsible for the COVID-19 pandemic. At the end of 2019, the World Health Organization (WHO) received reports of a cluster of pneumonia cases in China specifically in Wuhan. The cause was still unknow. Few weeks later, the cause was identified to be a novel coronavirus and was temporarily named “2019-nCoV”.^1^ In March 2020, there was a rapid increase in the number of cases worldwide which prompted the WHO to announce that the outbreak could be characterized as a pandemic. Globally, as of September 2021, there have been more than 230 million cases of COVID-19 and more than 4.5 million deaths of this virus.^1^

The SARS-CoV-2 is known to have an incubation period of 14 days after the exposure. The clinical presentation includes a wide variety of symptoms most commonly fever, cough, myalgias, and headache. The diagnosis is made on laboratory and imaging findings. The severity of COVID-19 infection ranges from asymptomatic and pre-symptomatic to mild, moderate, severe, and critical cases. The severity of illness is determined based on symptoms, oxygen saturation, and respiratory function. The management of COVID-19 is mainly symptomatic with supportive care while many antiviral and antimalarial medications have been used as part of the management as well.^2^

The pandemic had a significant impact on cancer care services worldwide. In the pandemic early phase, many procedures including surgeries, radiotherapies and systemic therapies were cancelled including these planned for patients with curable cancers as the risks of being exposed to COVID-19 infection were considered to outweigh the benefits early cancer diagnosis and treatment.^3^

By the end of 2020, two vaccines, Pfizer-BioNTech and Moderna/US NIAID, received the Emergency Use Authorization (EUA) and the process of distribution was commenced. The availability of the vaccine resulted in reducing the transmission of the virus and the number of COVID-19 positive cases. Since cancer patients belong to the vulnerable population, vaccination was needed to reduce their risk of infection. Furthermore, vaccinating patients and healthcare providers would make it safer for cancer patients to attend their hospital visits and receive their treatments.^4^

The aim of this article is to review the recent evidence and recommendations regarding COVID-19 vaccination in adults and adolescents above 12 years old with cancer.

## Vaccination in Cancer Population

Due to the negative changes in their immune system, cancer patients are at a higher risk of acquiring infections compared to the general population and infections in these patients often result in excess morbidity and mortality. This increased risk might be related to several causes including the cancer itself, immunosuppressive treatments, and malnutrition.^5^

Thus, cancer patients are recommended to be vaccinated based on a prespecified immunization schedule. The vaccine administration time varies depending on several factors such as the type of the vaccine. According to the Infectious Diseases Society of America (IDSA) clinical practice guideline for vaccination of the immunocompromised host, live attenuated vaccines should be given at least four weeks prior to the initiation of any immunosuppressive therapy and should not be administered during radiation or chemotherapy as this might trigger a vaccine-derived infection. Inactivated vaccines should be given at least two weeks prior to the immunosuppressive therapy, and be avoided during radiation and chemotherapy. Generally, the vaccines that use live attenuated virus or replicating viral vectored vaccines are contraindicated in Hematopoietic cell transplantation (HCT) or Chimeric antigen receptor (CAR) T-cell treated patients.^6,7^

## Safety and Efficacy of COVID-19 in Cancer Population

As of the January 2022, 10 COVID-19 vaccines were validated by the WHO and had received the Food and Drug Administration (FDA) EUAs. These vaccines are; Pfizer-BioNTech, Oxford/AstraZeneca, Serum Institute of India (Oxford/AstraZeneca formulation), Janssen (Johnson & Johnson), Moderna, Sinopharm (Beijing), Sinovac, Bharat Biotech, Novavax, Serum Institute of India (Novavax formulation). The EUA grants the use of the unapproved medical products in emergency situations when statutory criteria have been met, such that there are no adequate, approved and available alternatives to compensate until FDA finishes their detailed and lengthy approval process.^8^ Detailed list of approved vaccines summarized in Table 1.

**Table 1.** List of Vaccines Granted Emergency Use Authorization.

Vaccines	Emergency Use Authorization Approval Date	Vaccine Type	Food and Drug Administration Approval
Pfizer/BioNTech: Comirnaty®	31st December 2020	mRNA vaccine	
Oxford/AstraZenecaVaxzevria®	15th February 2021	(ChAdOx1-S ([recombinant])	
Serum institute of India (Oxford/AstraZeneca formulation) Covishield®	15th February 2021	(ChAdOx1-S ([recombinant])	
Janssen (Johnson & Johnson) Ad26.COV2. S®	12th March 2021	(Ad26.COV2-S [recombinant])	
Moderna:Spikevax®	30th April 2021	mRNA vaccine (nucleoside modified)	
Sinopharm (Beijing) Covilo®	07th May 2021	Inactivated vaccine (vero cell)	
SinovacCoronaVac®	01st June 2021	Inactivated vaccine (vero cell)	
Bharat BiotechCovaxin®	03rd November 2021	(Whole virion inactivated corona virus vaccine)	
NovavaxNuvaxovid®	20th December 2021	(SARS-CoV-2 rS [recombinant, adjuvanted])	
Serum institute of India (novavax formulation) COVOVAX®	17th December 2021	(SARS-CoV-2 rS protein nanoparticle [recombinant])	

On the 31st of December 2020, Pfizer-BioNTech (mRNA-BNT162b2) was granted the first EUA. It has been shown to be 95% effective against COVID-19 for fully vaccinated individuals after a week from the second vaccine.^9^ The safety profile for this vaccine was fairly acceptable across all populations and the most frequently reported events were fatigue and headache. FDA authorized the emergency use for Adolescents 12 through 15 years of age in May 2021 and for children 5 through to 11 years of age in October 2021.^10,11^ Pfizer-BioNTech (mRNA-BNT162b2) was later granted the first FDA approval on the 23rd of August 2021 and marketed as “Comirnaty” for the prevention of COVID-19 disease in individuals 16 years of age and older.

Moderna/US NIAID (mRNA-1273) was granted the EUA on December 18, 2020. The phase 3 clinical trial proved the vaccine to have an appropriate safety profile. Most events were mild to moderate while the severe events of injection site reaction, arthralgia, myalgia and headache were rare. The Moderna/US NIAID was shown to be 94.5% effective in COVID-19 infection prevention.^12^ On the other hand, according to the Vaccine Adverse Event Reporting System (VAERS), cases of myocarditis were reported in males’ adolescents and young adults usually within a week of the second dose.

However, recent Available data showed that the SARS-CoV-2 infection can cause myocarditis independent of the vaccine, hence the use of the vaccine was recommended as the benefit of the mRNA vaccine would outweigh the risk. Furthermore, it was noted that myocarditis and pericarditis following vaccination were generally mild and responded well to available treatments.^13^

The viral vector for the Oxford–AstraZeneca vaccine was engineered using ChAdOx1 vector to include the information that codifies for the wildtype SARS-CoV-2 Spike protein. The vaccine showed 64.1% efficacy after the first dose and 70.4% for fully vaccinated individual with no safety concerns as per the interim analysis of four trials across three continents.^14^ Oxford–AstraZeneca name was changed to “*Vaxzevria”* in European Medicines Agency (EMA) as well as the Medicines and Healthcare products Regulatory Agency in the UK and Australia. In India, the local version of “*Vaxzevria”* is produced by the Serum Institute of India (SII) under the name “Covishield”.

The first single-dose vaccine to secure EUA was JNJ 78436735/Ad26.COV2.S1 produced by Johnson & Johnson (J&J) pharmaceutic.^15^ The efficacy of this vaccine after at least 14 days post administration was 66.9% and the efficacy increased with time reaching 85.4% 28 days post dose. The safety profile of J&J vaccine was similar to the other vaccines with injection site reaction as the most reported local reaction while headache, fatigue, myalgia, and nausea are the most general side effects. On the other hand, post marketing surveillance showed some concerning data about the J&J vaccine. The Centers for Disease Control and Prevention (CDC) provided data of 47 cases (mainly women younger than 50 years old) of thrombosis with thrombocytopenia which is rare but clinically serious and potentially life threating condition. In addition, VAERS preliminary reported around 210 cases of Guillain-Barré Syndrome 2 weeks post J&J vaccine mainly in 50 years and older males.^16^

The first Chinese vaccine to be granted the emergency use was Wuhan Institute of Biological Products/China National Biotech Group-Sinopharm commonly known as Sinopharm. According to phase 1/2 studies, no serious adverse events were reported within 28 days post vaccination and all adverse effects reported were mild to moderate.^17^

The efficacy of the vaccine was measured by the neutralizing antibody geometric mean titers which was shown to have 100% seroconversion and was found in all participants on day 42. Participants received two doses: the first dose at day 0 and the second at day 21 or day 28.

A multi-center phase 3 trial showed an efficacy of 79% against symptomatic COVID-19 14 days or more post second dose.^17^

Sinovac was well tolerated and induced humoral responses against SARS-CoV-2. It was proven to be well tolerated and moderately immunogenic in healthy adults aged 18-59 years. According to PROFISCOV study, the incidence of Severe adverse effect was only .5% and all of them were determined to be unrelated to the vaccine. The rates of local adverse reactions (Vaccination site pain, swelling, pruritis, redness and induration) were statistically significant (<.0001) while the incidence of systemic adverse reactions was not statistically significant as the results crossed the line of significance (.3882).^18^ Sinovac received the EUA on the first of June 2021

COVAXIN^®^, India^’^s indigenous COVID-19 vaccine by Bharat Biotech which was developed in collaboration with the Indian Council of Medical Research (ICMR) and National Institute of Virology (NIV). COVAXIN is an inactivated virus vaccine taken as 2-dose vaccination regimen 28 days apart. In the phase 3 multicenter study, COVAXIN^®^ demonstrated 77.8% vaccine efficacy against symptomatic COVID-19 disease, while the efficacy against asymptomatic COVID-19 was 63.6%. 12% of the patients experienced common side effect and in less than .5% of them, h adverse events were serious.^19^

NVX-CoV2373 (Novavax) is a protein-based vaccine engineered from the genetic sequence of the first strain of SARS-CoV-2, the virus that causes COVID-19 disease.^19^ The efficacy of this vaccine was evaluated in two randomized, placebo-controlled, observer-blinded trials; a trial in the UK and another in the USA & Mexico.^19^ In the UK, the vaccine showed 89.7% efficacy.^20^ The PREVENT-19 trial in USA & Mexico with 25 452 participants showed 90.4% efficacy overall. It was generally well-tolerated and elicited a robust antibody response in both studies.^21^

To date, and apart from live attenuated virus or replicating viral vectored vaccines in general, there are no contraindications for the COVID-19 Vaccine among people with any solid tumors and across the broad range of therapies such as: Cytotoxic, radiation, hormonal, targeted and immunotherapy.^22^ Moreover, it’s also not contraindicated in people with hematologic malignancies receiving different regimens including Hematopoietic Stem Cell Transplant (HSCT) and CAR T cell therapy. It is worth mentioning that although the National Comprehensive Cancer Network (NCCN) recommended that cancer patient should be prioritized for the COVID-19 vaccines, there remains uncertainty regarding the mechanism of action and efficacies of these vaccines in cancer patients as most cancer patients were excluded from the vaccines’ clinical trials in view of the immunocompromised status.

To date on the immunogenicity of vaccine in cancer population is limited only on the measurement of post-vaccine titers to the viral spike protein. Primary data showed limited response in immunosuppressed population, these results warranted the NCCN to recommend a third dose for cancer population.

## Recommendations About COVID Vaccine in Cancer Population

The available evidence suggests that patients with cancer are at a greater risk for severe disease, intensive care admission and mortality from COVID-19 infection compared to the general population.^23^ The CDC declared that patients with certain medical conditions including cancer are liable to have severe illness from COVID-19. Such population are more likely to be hospitalized, need admission to the intensive care unit, require a ventilator, or die of the COVID-19 infection.^2^

As several COVID-19 vaccines are currently available, this high-risk population would benefit from receiving the vaccine to prevent COVID-19 infection. The Advisory Committee on Immunization Practices at the CDC prioritized patients with high-risk conditions including patients with cancer to be allocated for vaccination in phase 1c.^24^

The vaccine will provide immunity against the virus; hence, vaccinated patients will be less prone to COVID-19 infection and COVID-19 related complications. As per the WHO, vaccinated personnel have strong protection against COVID-19 related complications including serious illness, hospitalization, and death.^1^ The American Society of Clinical Oncology (ASCO) recommends offering COVID-19 vaccine to patients with cancer as long as they do not have any contraindication to the vaccine.^25^ Examples of contraindications to COVID-19 vaccine are severe allergic reaction after a previous dose of COVID-19 vaccine or immediate reaction of any severity to polysorbate or any components of the vaccine.^2^ However, recommendations about timing of vaccination varies depending on the type of cancer and cancer treatment. This section will cover the recent recommendations regarding providing COVID-19 vaccine to adults and adolescents with cancer. It should be noted that most of the recommendations are based on guidelines that were developed by professional personnel in the field.

### Recommendations Based on Cancer Type

#### Patients with Hematologic Malignancies

Vaccination in patients with hematologic malignancies depends mainly on patients’ immune system and is guided by eligibility criteria. Firstly, the host’s ability to mount a cellular and humoral immune response. If the host is not able to generate a fully protective immune response to the COVID-19 vaccine, such patients could have absented or attenuated response to the vaccine.^26^

Recent evidence showed that antibody response was highly variable in patients with multiple myeloma who completed the recommended two doses of vaccination. Thus, serological monitoring and personalized risk reduction measured might be intended in patients with multiple myeloma even after being fully vaccinated.^27^

Furthermore, in patients with Chronic Lymphocytic Leukemia, the antibody mediated response to the Pfizer-BioNTech vaccine was impaired due to both disease activity and treatment and these patients might benefit from serological tests after the second dose of the vaccine to assess the response to the vaccine.^28^

Secondly, the type of hematologic malignancy affects the timing of the vaccination. In most cases, newly diagnosed patients and patients scheduled for induction therapy should not delay the systemic therapy for vaccination purposes. Also, the vaccine should be given to patients during maintenance phase when they show evidence of hematopoietic count recovery.^29^ Disease specific recommendations are summarized in Table 2 according to the Memorial Sloan Kettering Cancer Center (MSK) COVID-19 vaccine guidelines.^29^ Thirdly, the type and timing of recent therapy which will be discussed later.

**Table 2.** Disease Specific Recommendations for Vaccination Time Based on Hematologic Malignancy Type.

Disease	Recommendation
B-cell acute lymphoblastic leukemia or T-cell acute lymphoblastic leukemia	- Induction therapy should not be delayed - Vaccine should be given during the maintenance phase when patient shows evidence of hematopoietic count recovery - Vaccine could be given during induction for patients on less intense regimens (eg, steroids + TKI)
Chronic lymphocytic leukemia	- Asymptomatic patients, B-cell depleting therapy could be held until one month after completion of vaccination - Symptomatic patients on small molecule therapy, vaccination should be held until 1 month after completion of treatment and once there is evidence of B-cell recovery^a^ - Symptomatic patients on chronic treatment, vaccination should be considered
Diffuse large B-cell lymphoma and other aggressive B-cell lymphomas	- Systemic induction therapy should not be delayed for newly diagnosed patients - Vaccine should be given after completion of therapy and once there is evidence of B-cell recovery^a^
Indolent lymphomas	- Asymptomatic patients, B-cell depleting therapy could be held until one month after completion of vaccination - Patients requiring systemic therapy should be treated with induction but without maintenance therapy, and vaccine could be given following completion of therapy and once there is evidence of B-cell recovery^a^
T cell lymphomas	- Therapy should not be delayed for newly diagnosed patients and patients with progressive disease for vaccination purposes - Vaccine could be given during induction therapy, preferably following count recovery
Lymphoma patients with relapsed or refractory disease	- Systemic therapy with the potential for therapeutic benefit should not be delayed for vaccination purposes
Myeloma	- There are no specific disease or treatment related contraindications for vaccination in patients with myeloma expect for patients on lymphodepleting therapy - Vaccine could be attempted in patients treated with lymphodepleting therapy once patient shows evidence of lymphocyte recovery as aligned with HSCT and cellular therapy guidelines
Acute myeloid leukemia	- Therapy should not be delayed for newly diagnosed patients for vaccination purposes - Vaccine should not be considered during induction remission phase - Vaccine could be given during consolidation therapy - Vaccine could be considered for patients with relapsed disease
Chronic myeloid leukemia	- Vaccine should be given for patients receiving TKIs (with or without remission)
Myelodysplastic syndromes	- Vaccine should be given for patients on observation or active therapy with hypomethylating agents
Myeloproliferative neoplasm	- Vaccine should be given for patients with essential thrombocythemia (ET), polycythemia vera, or myelofibrosis on observation or active therapy

Abbreviation: TKI, tyrosine kinase inhibitor.

^a^B-cell recovery: Absolute lymphocyte count ≥1.0 and B-cell count ≥50 cells/lymph by flow cytometry.

#### Patients with Solid Tumors

According to the MSK COVID-19 guidelines, patients with solid tumors should receive COVID-19 vaccine in absence of contraindications to the vaccine and being on active therapy is not a contraindication to the vaccine. This recommendation is applied to cytotoxic chemotherapy, radiation therapy, hormonal therapy, targeted therapies, immunotherapy, corticosteroids, and surgical management.^29^ These therapies should not be paused or delayed for vaccination purposes.^29^ On the other hand, although the optimal timing of vaccine administration for those patients is not established by the guidelines yet, some recommendations in certain circumstances were established and these are summarized in Table 3.

**Table 3.** Recommendations About Timing of Vaccination in Certain Circumstances.

Case	Recommendation
Patients planned for but not yet on immunosuppressive therapy	Vaccine should be given ≥2 weeks prior to initiation of therapy if feasible
Patients already on cytotoxic chemotherapy	Vaccine should be given in between chemotherapy cycles, and away from nadir phase
Patients completing cytotoxic therapy	Vaccine should be given after therapy is completed and nadir period is resolved
Patients undergoing cancer related surgery	No specific timing is recommended

### Recommendations Based on Cancer Treatment

#### Hematopoietic Stem Cell Transplant and Cellular Therapy Recipients

HCT and CAR T cell therapy recipients are at a greater risk of serious complications from COVID-19 infection. These patients are often immunosuppressed for months after the procedure due to several factors including conditioning regimens, maintenance therapies, immunosuppressive drugs, hypogammaglobinemia, or development of graft-versus-host disease (GvHD) in allogeneic HCT recipients and these factors can also affect the efficacy of the COVID-19 vaccine in such patients. On the other hand, there are no clinical trial data that determine the optimal vaccination timing.^30,31^ As per the NCCN guidelines, patients undergoing allogeneic transplantation or autologous transplantation or receiving cellular therapy should wait at least 3 months after the HCT/cellular therapy before being vaccinated.^32^

Moreover, the MSK issued a more detailed timing protocol according to the type of transplantation or cellular therapy as per Table 4 summary. Furthermore, the American Society for Hematology has provided some recommendations regarding vaccination timing in case the patient is scheduled for another therapy after transplantation or cellular therapy. Firstly, for patients requiring cytotoxic or B-cell depleting therapies after HCT or CAR T, COVID-19 vaccine could be given prior to therapy. Patients should wait at least 2 weeks after the completion of the second dose of the vaccine before receiving the intended therapy. Secondly, the vaccine should not be delayed in patients with hypogammaglobinemia due to poor B-cell function who require intra-venous immunoglobulins.

**Table 4.** Memorial Sloan Kettering Cancer Center Recommendations for Vaccination Time Based on Type of Transplantation or Cellular Therapy.

Treatment		Recommendation
Autologous HCT		Vaccination could be initiated 2-3 months post HCT
Allogeneic HCT	Conventional, no severe GVHD, no anti-CD20 antibodies	Vaccination could be initiated as early as 3 months post HCT
	Ex-vivo T-cell depleted and post HCT	Vaccination could be initiated around 6 months post HCT with confirmed presence of B cells (>50) and CD4^+^ T-cells (>100)
	Chimeric antigen receptor T cell therapy and receipt of antiCD20 antibodies	Vaccination may be initiated as early as 3 months if demonstrated human intravenous immunoglobulins (IVIG) independence and B-cell count ≥50
HSCT patients with GvHD		Patients could receive the vaccine since there is low chance that COVID-19 vaccine will exacerbate the GvHD

Abbreviations: HCT, Hematopoietic cell transplantation; GvHD, graft-versus-host disease; HSCT, Hematopoietic Stem Cell Transplant.

Finally, the vaccine should not be delayed in patients who become infected with SARS-COV-2 between the two doses, since there is no data suggesting presence of vaccine-associated enhanced disease or other serious adverse events. Hence the second dose of the vaccine could be given once symptoms resolved and isolation precautions discontinued.^30^ Moreover, The European Society for Blood and Marrow Transplantation recommended HCT recipients above 12 years old to be vaccinated against SARS-COV-2. Adolescents are recommended to receive either one of the two mRNA vaccines as these two are licensed vaccines for this population. The timing of the vaccination may be considered based on the rate of the transmission in the surrounding. The vaccination process could be initiated as early as three months after HCT if the risk of transmission is high while it could be logical to initiate it six months after HCT in case of low risk of transmission. Additionally, as for other vaccines, the procedure will most likely wipe out all immune memory. Thus, for patients who received the vaccine prior to HCT or cellular therapy, the vaccine should be discontinued, and vaccination process should be re-initiated again after the procedure.^31^

#### Chemotherapy

For patients on chemotherapy, receiving the vaccination is advisable as these patients are more vulnerable to COVID-19’s mortality. However, when given during an active treatment, it is preferable to be timed in-between cycles and away from nadir period. Vaccine’s side effects usually start 2-3 days post vaccine. If the side effects were present, the next chemotherapy cycle should be delayed. As mentioned before, NCCN recommends the vaccine to be taken when available for patient with solid tumor on chemotherapy.

However, in some circumstances, delaying the vaccination until the end of a very intensive chemotherapy treatment may be warranted, such as induction therapy for acute leukemia regimens.^29,32^

Notably, cancer patients receiving cytotoxic regimens are expected to have a lower antibody response compared to healthy individuals or cancer patients not on treatment. Hence, response to vaccine might not be optimal to illicit the response needed for protection from COVID-19.

#### Immunotherapy and Targeted Therapy

Although, there is no clear guidance on COVID-19 vaccination for all types of immunotherapy and targeted therapy patients, some therapies have some recommendations and points of concerns presented in the following paragraphs.

##### AntiCD20 Therapy

CD20 is a membrane-embedded surface molecule which plays a role in the development and differentiation of B-cells into plasma cells. CD-20 is expressed on B-lymphocytes and targeting these cells would reduce the vaccination induced humoral response and antibody production. Hence, efforts have been made to develop appropriate recommendations for the use of COVID-19 vaccines in patients on anti-CD20 targeted therapy.

**Figure 1.**
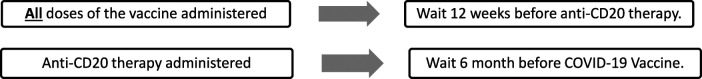
COVID-19 vaccination timing with respect to anti-CD20 therapy.

Previous studies exploring vaccinations in patients on rituximab (a known anti-CD20) showed that delaying the rituximab treatment for at least 12 weeks after administering the vaccine would be recommended to mount an appropriate immune response.^33^ However, If anti-CD20 was administered first, its recommended to wait for at least 6 months before the administration of the vaccine. (Figure 1) Despite of the recommendation of 6 months waiting before vaccination and in view of variations in the repopulation of B-cell post therapy, it might be appropriate to measure the B-cell populations after 3 months to assess the feasibility of early administration of the vaccine while taking in consideration other factors such as co-morbidities, infection and status of disease.^33^ Finally, it’s important to mention that experts are advising against any delay of the anti-CD20 therapies to administer the vaccine in high risk of relapse patients and patients with active disease

##### Targeted Therapy

The targeted therapies can be divided into tyrosine kinase inhibitors such as erlotinib, sunitinib, and imatinib and monoclonal antibodies (Mabs) such as trastuzumab. Although the normal immunosuppression effect of these drugs would predict an impaired immune response to the vaccines, this might not be the case as it was noted that antibody response following influenza vaccine in these populations where comparable to normal population.^34^

NCCN guidelines recommends the vaccine administration when available (providing no contraindications) to patients receiving targeted therapies as they are considered clinically extremely vulnerable group to develop serious COVID-19 related complications and severe acute respiratory syndrome.^32^

##### Immune Checkpoint

In multiple vaccine studies, treatment with immune checkpoint inhibitors such as atezolizumab, nivolumab and pembrolizumab was shown to be feasible with no significant detrimental effects on the sero-protection status. Thus, the administration of the COVID-19 vaccine to patients treated with immune checkpoint inhibitors is expected to mount an appropriate response. On the other hand, vaccinating patients treated with immune checkpoint inhibitors raises the concern of increase incidence of immune-related adverse events (irAEs) and the overload of the immune system that might trigger cytokine storm that, in turn, could lead to severe COVID-19 mortality and morbidity. However, this notion was refuted by several studies including a retrospective report on the incidence of irAEs in cancer patients treated with pembrolizumab who received the influenza vaccine and had a lower incidence of irAEs of any grade compared to the nonvaccinated group.^35^

Regarding the timing of the vaccine, the UK Chemotherapy Board recommends the administration of vaccine to be toward the end of the cycle when blood count is recovered but to avoid on days of chemotherapy administration.^36^ Other recommendations regarding immunotherapy and targeted therapies are mentioned in Table 5.^36^

**Table 5.** Recommendation Regarding COVID-19 Vaccination Timing Based on Agents.

Agents	Recommendation
1**.** Monoclonal antibody (+chemotherapy) 2. Immunotherapy (+chemotherapy) 3. Immunomodulatory 4. Proteasome inhibitor 5. CDk4/6 inhibitors	Toward the end of the cycle when blood count is recovered but to avoid on days of chemotherapy administration
1. Monoclonal antibodies 2. Immunotherapy 3. Small molecule protein kinase inhibitors 4. PARP inhibitors	To be administered when available with no contraindications, providing an appropriate/normal full blood count

#### Radiotherapy

Radiation for cancer patients usually targets a large surface area and hence may affect the bone morrow. In other vaccines studies, the radiation did not alter the immune system’s response to the vaccines and therefore patients on radiation were encouraged to be vaccinated. According to the American Society for Radiation Oncology, cancer patients who are actively receiving radiation therapy are encouraged to consult their oncologist for the vaccination timing, location of the injection and any other individual considerations relevant to them.^37^ This is also emphasized in the NCCN guidelines in which patients under radiation therapy are encouraged to take the vaccine as soon as its available to them.^32^

### Recommendations Regarding the Third Dose of the COVID-19 Vaccines

Recently, FDA authorized additional vaccine dose for certain immunocompromised individuals. This only applies to mRNA COVID-19 vaccines. A third dose has been recommended for immunocompromised patients since the current data suggests that those patients can have impaired immune responses to vaccination. According to the CDC, a third dose is recommended for patients who are receiving active cancer treatment, high-dose corticosteroids or other immunosuppressive therapy or received a stem cell transplant within the last 2 years. The third dose should be administered at least 4 weeks after the second dose of the vaccine. Also, if possible, the third dose should be of same mRNA vaccine the patient received previously.^38^

Similarly, The NCCN provides guidelines regarding administration of a third dose of the vaccine in cancer patients and recommends that patients selection should be based on underlying cancer, therapy, and other immunocompromising conditions. Firstly, patients with active hematologic malignancies should be offered a thirds dose of the vaccine even if they are not on active cancer therapy.

Secondly, patients with solid tumor malignancies who received cancer therapy within 1 year of the initial dose of the vaccine should receive a third dose. This recommendation includes patients newly diagnosed with cancer or currently receiving active therapy for cancer. However, this recommendation does not apply to patients with non-melanoma skin cancers or patients with superficial mucosal lesions treated solely with local therapy. Thirdly, a third dose is recommended for patients undergoing allogenic transplantation and actively receiving immunosuppressive therapy and patients with GvHD. Moreover, patients who are ≤2 years post-HSCT should also be prioritized for the third dose. Fourthly, cancer patients who have other concurrent immunocompromising conditions should also receive a third dose of the vaccine. This would also include patients treated with systemic corticosteroids and other immunosuppressive agents. Finally, patients who became infected with COVID-19 infection after their initial vaccine doses should receive a third dose. With regard to the timing of the third dose administration, it is recommended to delay it for at least 28 days after completion of the initial two vaccine doses and after documentation of clearance of SARS-CoV-2 virus. It is not recommended to use antibody titer to decide whether the patient should receive the third dose or not the NCCN.^32^

## Safety of the Current Available Vaccines

In general, COVID-19 vaccines are considered to be safe and effective. However, some side effects have been reported in the general population. The most commonly reported acute side effects of the COVID-19 vaccines are injection site pain, muscle pain, fatigue, headache, fever, and chills.

These side effects are more likely to occur in younger population and after the second dose of the vaccine.^39^ These are short-term side effects and usually resolve within few days after receiving the vaccine. Post vaccination, drinking plenty of fluids and taking medications including paracetamol may help to alleviate fever and pain. Nonetheless, the current guidelines do not recommend pre-medication before the vaccine to reduce the side effects.^39^

In cancer patients, the safety of COVID-19 vaccination has been evaluated in recent studies. In a study of Pfizer-BioNTech vaccine in cancer patients who are treated with immune checkpoint inhibitors compared to a matched heathy control group, the side effect profile was similar in both groups except for the muscle pain. The study revealed that muscle pain was the only side effect that was reported more in the cancer group. The difference between the two groups was statistically significant. Interestingly, this study did not report any immune related side effects nor any exacerbation of pre-existed immune related side effects as a consequence to the vaccine.^40^ One important side effect that should be monitored in cancer patients is the axillary adenopathy. Some vaccine recipients, mRNA COVID-19 vaccines mainly, reported developing swelling or tenderness of the lymph nodes under the arm in which they got the injection. This is particularly important for patients with breast cancer and hence it is recommended to give the vaccine in the arm opposite to the site of the breast cancer.

However, if these patients developed post-vaccination axillary adenopathy, it is advisable to perform a short term follow up exam in 4-12 weeks following the second vaccine dose and if the adenopathy persists, a lymph node sampling to exclude breast and non-breast malignancy should be considered.^41^

On the other hand, the serious acute side effects of the COVID-19 vaccine are very rare and, to date, only two were reported; anaphylaxis and thrombosis with thrombocytopenia syndrome (TTS). Between December 2020 and January 2021, the rate of anaphylaxis post mRNA COVID-19 vaccines in the United States was reported to be 4.7 cases per million doses administered for Pfizer- BioNTech vaccine and 2.5 cases per million doses administered for Moderna vaccine.^42^ Based on this, it is recommended to monitor all vaccine recipients for 15-30 minutes after each vaccine dose for any symptoms of severe allergic reaction including generalized urticaria, significant tongue or lips swelling, or respiratory distress.

On the other hand, TTS is vaccines type specific as it is reported in the Johnson & Johnson’s Janssen COVID-19 Vaccine recipients. The risk of developing TTS is rare but more likely to occur in women below 50 years old. According to the CDC, the rate of TTS in women between 18 and 49 years old is about 7 per 1 million vaccinated women. In women with age of 50 years and older and men of all ages the risk is rarer. Therefore, women younger than 50 years old should be informed about this rare risk and it is advisable to receive other COVID-19 vaccines when possible.^39^

Myocarditis and pericarditis have been reported in 1491 individuals after receiving mRNA COVID-19 vaccine (Pfizer-BioNTech or Moderna) and were more common among male adolescents and young adults. However, The CDC is still investigating the relationship between these reported cases and COVID-19 vaccine.^39^

Finally, no long-term or chronic side effects have been reported from COVID-19 vaccines yet. However, long-term studies on vaccine recipients are needed to evaluate the long-term complications from these vaccines.

## Gaps in Current Evidence

### Studies Were Done Faster to Get the Emergency Use Authorization Approval

Although it is known that the procedure followed by the FDA to grant EUA has been a rigorous process that analyzed the vaccine’s safety and effectiveness and required a conclusion that potential benefits outweigh any potential risks, it is still perceived lightly due to the quantity and quality of studies needed to receive an EUA.^8^

The lengthy comprehensive process to achieve the FDA approval has always given the public trust in the pharmaceutical products. Hence, with almost all vaccines granted only an EUA, the public seems to be wary about them and reluctant to getting vaccinated.

### Population in the Studies

For safety reasons, special populations, especially cancer patients, have always been in the exclusion criteria for the per-marketing stages of most of the pharmaceutical products.^43^ It is understandable to spare the vulnerable population the first trial in normal circumstance.

Cancer patients are suffering from decline immune response due to the nature of the disease such as hematological malignancies or due to the administration of medications that depletes the immune response.^44^ Therefore, its speculated for subset of the recipients to have poor immune response to the vaccine and might need different dosing regimen than the one described in trials for healthy population.

### Recommendations Are Based on Other Vaccines in Cancer Patients

Many of the recommendation mentioned in this paper were collected from different guidelines and articles discussing the vaccination in other than COVID-19 setting. In the absence of cancer patient’s inclusion in the trials, the experts extrapolated the recommendation from other studies done on other vaccines. Although this might have been the only available data to follow, it certainly does not provide the most specific and personalized guidance in regard of the COVD-19 Vaccine. SARS-CoV-2 is a novel unique virus that invaded the globe in a few months with its high transmission rate and long incubation period.

Predicting a vaccine that target such a novel unique virus using other vaccines that target other viruses is not the most appropriate optimal approach. And therefore, the ASCO and the European Society of Medical Oncology calling for action to make cancer patients a high priority in the setting of COVID-19 Vaccine.^45^

## Conclusion

The Covid-19 pandemic has impacted all sectors of life with the highest impact on the healthcare. After the development of the vaccines and the issuing of EUAs, there was a glimpse of hope that life could go back to normality. However, for cancer population the situation is a complicated one due to the exclusion from vaccine approval trials and the complexities of the treatment regimen and body status. In this review we tried to collect current data regarding the use of different Covid-19 vaccines in different disease and treatment settings. Cancer patients are among the high risk for Covid-19 complications and mortality and according to guidelines and recommendations from different institutions, they should be prioritized for the vaccine.

The general consensus is for the vaccine to be given when available after individual considerations to immune system state and urgency of treatment. Covid-19 vaccines were proven to be safe with mild to moderate adverse events.

Although Evidence and reports are trying to keep up with daily changes of recommendations regarding COVID-19, there are still gaps that need to be addressed. Due to the fast track that vaccines had followed to be granted the EUA, the public are still wary and reluctant to get the vaccination. Special population-including cancer patients- were excluded from the approval trials for their safety. However, due to the complexity of the cancer pathophysiology, it is hard to speculate dosing and regimen using healthy populations trials data. Last of all, most of the cancer patients’ recommendations regarding the COVID-19 vaccines are extrapolation from other vaccine studies.

In order to fill the current knowledge gaps, further studies are needed to assess different diseases states and treatment plans. Since the vaccines have been showing some promising safety results, efforts should be put toward observational studies for cancer patients to have a better safety and efficacy profile.
